# State of affairs regarding targeted pharmacological therapy of cancers metastasized to the brain

**DOI:** 10.1007/s10143-022-01839-8

**Published:** 2022-07-29

**Authors:** Hans-Jakob Steiger, Kathrin Vollmer, Susanne Rogers, Lucia Schwyzer

**Affiliations:** 1 Department of Neurosurgery, Kantonsspital Aarau, Aarau, Switzerland; 2 Klinik Für Neurochirurgie, Neurozentrum, Kantonsspital Aarau, Tellstr. 25, CH-5001 Aarau, Switzerland; 3 Department of Neurosurgery, University Hospital Düsseldorf, Düsseldorf, Germany; 4 Division of Oncology, Hematology and Transfusion Medicine, Kantonsspital Aarau, Aarau, Switzerland; 5 Radio-Oncology-Centre KSA-KSB, Kantonsspital Aarau, Aarau, Switzerland

**Keywords:** Targeted therapy, Personalized therapy, Brain metastasis, Advanced cancer

## Abstract

In 1999 a visionary short article by The Wall Street Journal writers Robert Langreth and Michael Waldholz popularized the new term “personalized medicine,” that is to say, the targeting of drugs to each unique genetic profile. From today’s perspective, targeted approaches have clearly found the widest use in the antineoplastic domain. The current review was initiated to review the progress that has been made regarding the treatment of patients with advanced cancer and brain metastases. PubMed was searched for the terms brain metastasis, brain metastases, or metastatic brain in the Title/Abstract. Selection was limited to randomized controlled trial (RCT) and publication date January 2010 to February 2022. Following visual review, 51 papers on metastatic lung cancer, 12 on metastatic breast cancer, and 9 on malignant melanoma were retained and underwent full analysis. Information was extracted from the papers giving specific numbers for intracranial response rate and/or overall survival. Since most pharmacological trials on advanced cancers excluded patients with brain metastases and since hardly any information on adjuvant radiotherapy and radiosurgery is available from the pharmacological trials, precise assessment of the effect of targeted medication for the subgroups with brain metastases is difficult. Some quantitative information regarding the success of targeted pharmacological therapy is only available for patients with breast and lung cancer and melanoma. Overall, targeted approaches approximately doubled the lifespan in the subgroups of brain metastases from tumors with targetable surface receptors such as *anaplastic lymphoma kinase* (ALK) fusion receptor in non-small cell lung cancer or *human epidermal growth factor receptor 2* (HER2)–positive breast cancer. For these types, overall survival in the situation of brain metastases is now more than a year. For receptor-negative lung cancer and melanoma, introduction of immune checkpoint blockers brought a substantial advance, although overall survival for melanoma metastasized to the brain appears to remain in the range of 6 to 9 months. The outlook for small cell lung cancer metastasized to the brain apparently remains poor. The introduction of targeted therapy roughly doubled survival times of advanced cancers including those metastasized to the brain, but so far, targeted therapy does not differ essentially from chemotherapy, therefore also facing tumors developing escape mechanisms. With the improved perspective of patients suffering from brain metastases, it becomes important to further optimize treatment of this specific patient group within the framework of randomized trials.

## Introduction

In 1999 a visionary short article by The Wall Street Journal writers Robert Langreth and Michael Waldholz popularized the new term “personalized medicine” to mean the targeting of drugs to each unique genetic profile [43]. In the beginning, the idea of assembling a catalogue of the biological diversities was intended to find optimal therapies for individual genetic varieties of diseases with an inherited component. The term personalized medicine was quickly in everybody’s mouth, but alternative expressions such as individualized and targeted medicine were introduced. Pharmaceutical companies subsequently made huge investments to develop targeted drugs for a variety of congenital, neoplastic, degenerative, and inflammatory diseases. Malignancies were not the primary idea of personalized medicine, but it was the area with the widest applications during the coming years. Not only the individuality of the patient, but also the individuality of the tumors was subsequently targeted. Due to the instability of the tumor genome, the field of oncology is certainly one of the biggest challenges for personalized medicine.

Originally Langreth and Waldholz certainly thought of specifying therapies according to the genomic individuality of patients, therefore the term personalized medicine. Since the concept was most successful with cancer treatment and the genomic properties of the cancer gene became the target, the term for the concept had to be adapted. The terms precision or targeted therapy are used today for therapies focusing on the specific properties of individual cancers.

Management of CNS metastases primarily involves local therapy including stereotactic radiosurgery, whole-brain radiotherapy, and surgery [71]. Long-term outcome then depends on systemic control of the underlying disease and prevention of intracranial recurrences. Adjuvant therapies with proven intracranial activity are therefore critical.

In principle, therapeutic approaches can be targeted to any aspect of the complex cellular machinery, i.e., *protein kinase B* (PKB), also known as AKT, *AMP-activated protein kinase* (AMPK), apoptosis signaling, hormone signaling, *Janus kinase-signal transducer and activator of transcription* (JAK-STAT) signaling, *mitogen-activated protein kinase* (MAPK) signaling, *mammalian target of rapamycin* (mTOR) signaling, *nuclear factor kappa-light-chain-enhancer of activated B cells* (NF-κB) pathway, *notch* signaling, *p53* signaling, *transforming growth factor β* (TGF-β) signaling, *Toll-like receptor* (TLR) pathways, *vascular endothelial growth factor* (VEGF) signaling, w*ingless-related integration site* (WNT) signaling, and more. Interference certainly appears most promising in cases of pathway deficits or hyperactivity because of mutations or genetic defects.

From today’s perspective, targeted approaches have found clearly the widest use in the antineoplastic domain. Here, targeted approaches encompass hormone therapy, signal transduction blockade, gene expression modulation, and induction of apoptosis, anti-angiogenetic medication, immunotherapies, vaccines, and monoclonal antibodies carrying toxic substances (https://www.cancer.gov).

*Anti-hormone therapy* slows or stops the growth of hormone-sensitive tumors that require specific hormones. Production of hormones in the body is stopped or the action of hormones is blocked. Anti-hormone therapy is important for the treatment of hormone sensitive prostate and breast cancer.

**Fig. 1:**
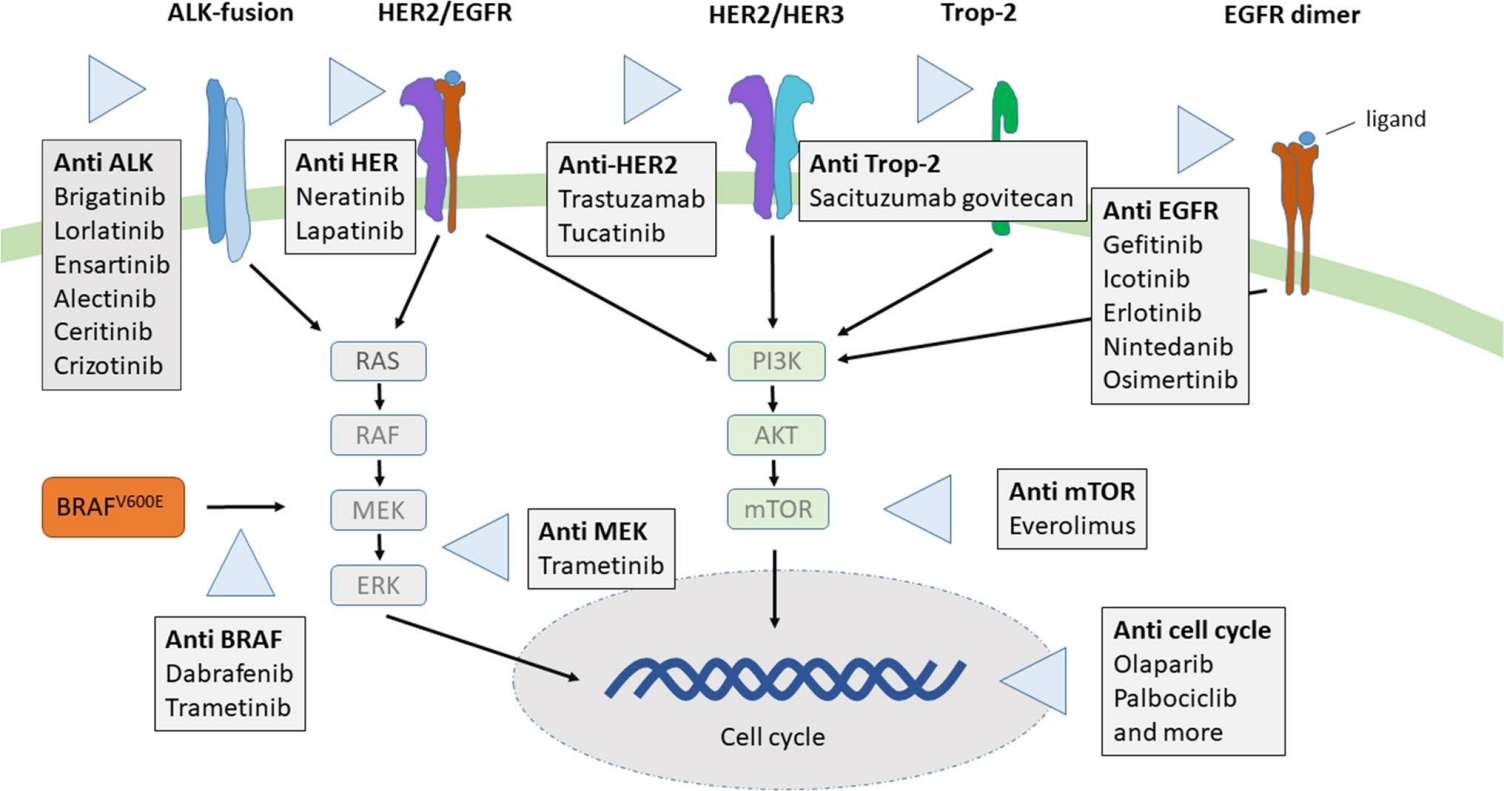
Main surface receptors and signal pathways currently targeted for breast and lung cancer, and melanoma, as well as the therapeutically used blocking antibodies. ALK, anaplastic lymphoma kinase; HER2, human epidermal growth factor receptor 2, also known as ERBB2, erb-b2; EGFR, epidermal growth factor receptor; Trop-2, trophoblast cell surface antigen 2

*Signal transduction inhibitors* block the activity of molecules involved in signal transduction, the process by which cells respond to environmental signals (Fig. 1). When a cell receives a particular signal, the signal is transmitted into the cell through a series of biochemical reactions that ultimately trigger the appropriate response. In some cancers, malignant cells internally generate a proliferation signal even without an external growth signal. Signaling inhibitors interrupt this inappropriate signaling.

*Gene expression modifiers* adapt the effect of peptides involved in the regulation of gene expression.

*Apoptosis inducers* cause apoptosis in cancer cells. Malignant cells have developed strategies to avoid apoptosis. Apoptosis-inducing agents can circumvent these strategies and cause cancer cells to die.

*Angiogenesis inhibitors* prevent the growth of new blood vessels in the tumor and therefore limit tumor growth. Treatments that inhibit angiogenesis can stop tumor growth. Some targeted therapies that inhibit angiogenesis block the action of VEGF, while others bind to the VEGF receptor or other molecules involved in angiogenesis.

**Fig. 2:**
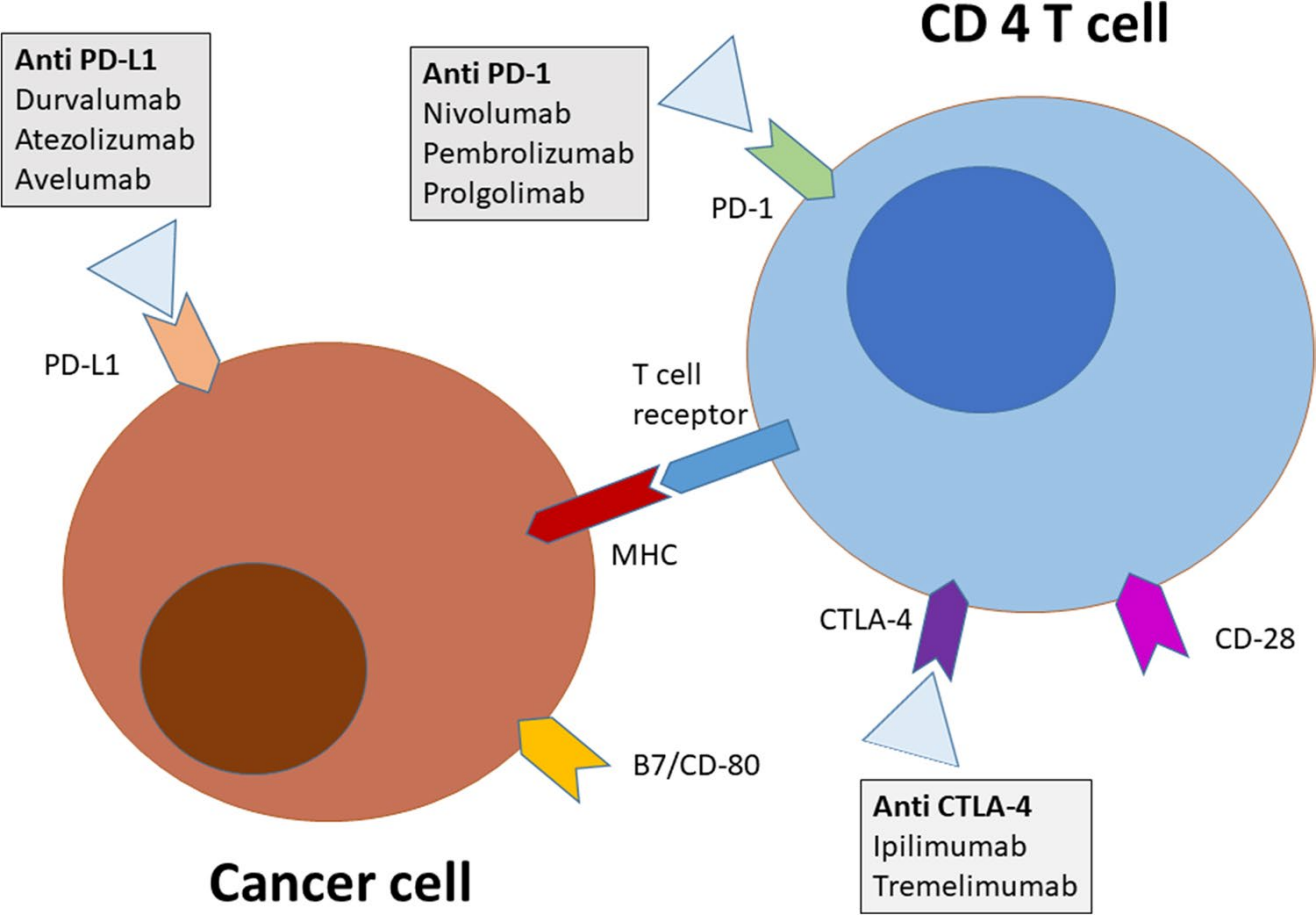
Main cell signal pathways, targets and therapeutic blocking antibodies, currently used in immune cancer therapy. PD-1, programmed cell death protein 1; PD-L1, programmed death-ligand 1; CTLA-4, cytotoxic T-lymphocyte-associated protein 4; MHC, major histocompatibility complex

*Immunotherapy* helps the immune system destroy malignant cells. Certain monoclonal antibodies bind to immune cells and help them avoid the escaping mechanisms developed by the tumor cells (Fig. 2). Other monoclonal antibodies support the effect of cytotoxic T-cells by binding to specific molecules on the surface of cancer cells.

*Antibody–drug conjugates* can specifically kill cancer cells. Once the antibody binds to the target cell, toxic molecules attached to the antibody, such as radioactive or toxic chemicals, are taken up into the cell, ultimately killing the cell. For cells that are not the target of antibodies, i.e., the majority of somatic cells, toxins have a much smaller effect.

Within the field of primary brain tumors, although prognostic biomarkers have been identified and a number of clinical phase I–II trials were conducted, so far no promising therapy has emerged for glioblastoma [85]. Bevacizumab, an antiangiogenic medication has been approved as symptomatic treatment for glioblastoma although studies do not indicate longer survival. Everolimus, an mTOR inhibitor, is approved for subependymal giant cell astrocytoma in adults and children aged 1 year or older who have tuberous sclerosis and are not able to have surgery. Moreover, the FDA has approved belzutifan for patients with *von Hippel-Lindau* (VHL) disease–associated tumors. VHL has a high incidence of renal cell carcinoma and other cancerous and non-cancerous tumors owing to VHL gene inactivation and constitutive activation of the transcription factor *hypoxia-inducible factor 2α* (HIF-2α) [39]. Larotrectinib, an inhibitor of *neurotrophic receptor tyrosine kinase* (NTRK), was recognized as an orphan drug by the US Food and Drug Administration in 2015 and later in Europe and was approved for the treatment of metastatic solid tumors with an NTRK fusion protein [18]. Finally, the *rat fibrosarcoma* (RAF) inhibitors dabrafenib plus trametinib showed clinically meaningful activity in patients with BRAF V600E mutation-positive high- and low-grade glioma, with a safety profile consistent with that in other indications [81]. BRAF V600E mutation appears to be also a critical driver mutation for other intra- and extracranial primary tumors, such as papillary craniopharyngioma and others. At the time of writing the FDA just granted approval of the BRAF inhibitors dabrafenib trametinib for the treatment of adult and pediatric patients 6 years of age and older with unresectable or metastatic solid tumors with BRAF V600E mutation who have progressed following prior treatment and have no satisfactory alternative treatment options (https://www.novartis.com/news/media-releases/novartis-tafinlar-mekinist-receives-fda-approval-first-tumor-agnostic-indication-braf-v600e-solid-tumors).

From the neurological and neurosurgical perspective, most progress regarding the targeted approach has been made in the field of metastatic brain tumors. Here, the targeted approach for the intracranial pathology and the systemic disease led to substantial improvement of survival in some cancer types. Although management of CNS metastases primarily involves local therapy including stereotactic radiosurgery, whole-brain radiotherapy, and surgery, long-term outcome then depends on systemic control of the underlying disease and prevention of intracranial recurrences. In some situations with proven intracranial efficacy, primary systemic therapy is now also an option, especially for asymptomatic lesions. Adjuvant therapies with proven intracranial activity are therefore critical. However, firm data from randomized controlled trials are available only for brain metastases from the most common types of cancers leading to intracranial dissemination, lung and breast cancer, and melanoma. A further complication is the fact that patients with brain metastases were excluded in most randomized trials. The purpose of the current review was to summarize the state of evidence regarding targeted adjuvant therapy in these types of brain metastases.

## Methods

A PubMed search was done with the search terms: brain metastasis, brain metastases, or metastatic brain in the Title/Abstract. Selection was limited to randomized controlled trial (RCT) and publication date January 2010 to February 2022.

The search yielded 172 hits. The abstracts were screened, and publications not concerned with targeted therapies or corresponding to reports of non-pharmacological interventional studies, and papers not reporting outcome data, were eliminated. Fifty-one papers on metastatic lung cancer, 12 on metastatic breast cancer and 9 on malignant melanoma were retained and underwent full analysis.

## Results

### Breast cancer

Most trials investigating drug therapy for advanced cancer were restrictive regarding inclusion of patients with brain metastases. These patients either were excluded entirely or, if not, limited to brain metastases that had been treated and proven stable for defined periods.

Among the selected reports on brain metastases, 9 contained some specific information regarding the outcome of patients with brain metastases (see Table 1). Most targeted adjuvant concepts address *human epidermal growth factor receptor 2* (HER2)–positive tumors. The overall outlook for HER2 + tumors remains substantially better than for triple-negative breast cancers, that is, tumors neither expressing estrogen nor progesterone receptors nor HER2. Although endocrine therapy using tamoxifen or aromatase blockers is a firm part of hormone receptor–positive breast cancer, no randomized studies could be found addressing the effect of these adjuvant therapies for the situation of advanced breast cancer with brain metastases.

**Table 1:** Metastatic breast cancer—pertinent studies allowing some prognostic assessment for patients with brain metastases

Year	Author	Drug/combination	Action	Control	1st/ 2nd line	PFS (experimental)	OS (experimental)	PFS (control)	OS (control)	Intracranial response (experimental)	Intracranial response (control)	OS with brain metastasis (experimental)	OS with brain metastasis (control)	Comments
2022	Cortes [12]	Trastuzumab-deruxtecan	Anti-HER2	Trastuzumab emtansine	2nd	25.1	> 60	7.2	ca.60					AB-toxin, HER2 + , only asympt. BM allowed
2021	Curigliano [15]	Tucatinib or trastuzumab and capecitabine	Anti-*HER2*	Placebo + trastuzumab and capecitabine	2nd	7.6	24.7	4.9	19.2			18.8	11.4	OS of BM estimated
2021	Hurvitz [34]	Neratinib + capecitabine	Anti-pan-HER	Lapatinib + capecitabine	1sr/2nd							16.4	15.4	Sub-analysis from NALA trial
2021	Bardia [5]	Sacituzumab govitecan	Anti-Trop-2	Various chemotherapy	2nd	4.8	11.8	1.7	6.9	3%	0%	6.8	7.5	Triple negative BC, ASCENT trial, BM data estimated
2020	Seligmann [65]	Lapatinib + capecitabine	Anti-HER2	Trastuzumab + capecitabine	2nd					25%	71%	12	> 12	LANTERN phase II focus only on BM, OS estimates
2020	Lin [46]	Tucatinib, trastuzumab + Capecitabine	Anti-HER2	Trastuzumab + capecitabine	2nd	9.9	18.1	4.2	12	47%	20%	18.1	12	HER2CLIMB sub-analysis for BM, PFS refers to CNS
2019	Iwata [36]	Atezolizumab + nab-paclitaxel	Anti-PD-L1	Placebo + nab-paclitaxel	1st	7.2 (brain 4.9)	21.3	5.5 (brain 4.4)	17.6					Triple negative BC, sub-analysis from IMpassion130 trial, only asympt. BM allowed
2018	Takano [72]	Trastuzumab plus capecitabine	Anti-HER2	Lapatinib + capecitabine	2nd	6.1	31	7.1	50			18	30	ATTAIN, OS BM estimated
2015	Cortes [14]	Afatinib + vinorelbine	Anti-HER2	Investigator choice	2nd	3	12	4	12	10%	30%	12	12	small study focused on BM, high toxicity of study medication
2015	Perez [57]	Etirinotecan pegol	anti topoisomerase-I	Investigator choice	2nd/3rd	3	12.4	3	10.3			10	4.8	not limited to receptor profile

*BC* breast cancer; *BM* brain metastasis; *PFS* progression-free survival in months; *OS* overall survival in months; *HER2* human epidermal growth factor receptor 2; *MEK* mitogen-activated extracellular signal-regulated kinase; *PD-L1* programmed cell death ligand 1

Regarding systemic efficacy, Cortés et al. recently reported results of second-line treatment of metastatic HER2 + cancer with trastuzumab-deruxtecan, an antibody drug conjugate in comparison with trastuzumab emtansine. In both arms, outcome was much better than in comparable trials before. Treatment with trastuzumab-deruxtecan provided a systemic progression-free survival (PFS) of 25.1 months and an overall survival (OS) of more than 60 months (DESTINY-Breast03) [12]. Brain-specific outcome data are not yet available.

Curigliano et al. reported the final results of the HER2CLIMB trial comparing tucatinib or placebo, in combination with trastuzumab and capecitabine as second-line treatment of advanced HER2 + breast cancer [15, 46]. Median duration of OS for all patients was 24.7 months for the tucatinib combination group versus 19.2 months for the placebo combination group. Median duration of PFS was 7.6 months for the tucatinib combination group versus 4.9 months for the placebo combination group. Regarding patients with brain metastasis, estimated OS for trastuzumab and capecitabine was 18.8 versus 11.4 months. Objective intracranial response rate was also higher in the tucatinib arm (47.3%) versus the control arm (20.0%, *P* = 0.03).

Hurvitz et al. reported efficacy of neratinib plus capecitabine compared to lapatinib plus capecitabine in the subgroup of patients with central nervous system involvement from the NALA Trial on metastatic HER2 + breast cancer [34]. Patients with treated or untreated asymptomatic or stable brain metastases were eligible. Eighty-one of 101 had received prior CNS-directed radiotherapy and/or surgery. In the CNS subgroup, mean PFS was 7.8 months in the neratinib plus capecitabine group versus 5.5 months in the control arm, and mean OS was 16.4 versus 15.4 months. At 12 months, the cumulative incidence of progressive CNS disease was 26.2% versus 41.6%, respectively. In patients with target CNS lesions at baseline, confirmed intracranial objective response rates were 26.3% and 15.4%, respectively.

Summarizing current results for patients with brain metastases from HER2 + breast cancer, it can be deduced that current treatment strategies achieve an overall survival of more than 1 year from the time of treatment initiation for tumor dissemination including the brain.

Regarding triple-negative breast cancer, the prognosis is clearly worse and fewer data are available. Recently, Bardia and coworkers reported on 468 patients with or without brain metastases from triple-negative breast cancer who were randomly assigned to receive the *trophoblast cell surface antigen 2* (Trop-2) inhibitor sacituzumab govitecan or chemotherapy [5]. The median age was 54 years; all the patients had previously been exposed to taxanes. The median progression-free survival was 5.6 months and 1.7 months with chemotherapy. The median overall survival was 12.1 months with sacituzumab govitecan and 6.7 months with chemotherapy. The percentage of patients with an objective response was 35% with sacituzumab govitecan and 5% with chemotherapy. Sub-analysis of 61 patients with stable brain metastasis showed limited intracranial activity with an objective response rate (ORR) of 3% with sacituzumab govitecan versus 0% with chemotherapy [17]. The median PFS was 2.8 months with sacituzumab govitecan and 1.6 with chemotherapy. Median OS was 6.8 months with sacituzumab govitecan and 7.5 months with physician’s choice of treatment.

Immune checkpoint inhibitors were also trialed for these difficult to treat receptor negative breast cancers [13, 36, 63]. In the IMpassion130 trial, a small benefit for atezolizumab combined with nab-paclitaxel compared to placebo plus paclitaxel was shown for patients with advanced triple-negative breast cancer [36]. Sub-analysis indicated an intracranial PFS of 4.9 months versus 4.4 months. The benefit of immune checkpoint blockade for patients with brain metastases remains to be confirmed.

### Melanoma

For disseminated melanoma, immune checkpoint inhibitors have become the mainstay of treatment, although some efficacy has also been shown for the anti proto oncogene *B-Raf* (BRAF) agents dabrafenib, trametinib, and other drugs in BRAF mutated tumors [2, 3, 16, 19, 20, 22, 28, 30, 44, 47, 49, 51, 60, 73, 79, 80, 83]. The pertinent studies allowing some appreciation of intracranial efficacy are summarized in Table 2.

**Table 2:** Metastatic melanoma—pertinent studies allowing some prognostic assessment for patients with brain metastases

Year	Author	Drug/combination	Action	Control	1st/ 2nd line	PFS (experimental)	OS (experimental)	PFS (control)	OS (control)	Intracranial response (experimental)	Intracranial response (control)	OS with brain metastasis (experimental)	OS with brain metastasis (control)	Comments
2021	Tjulandin [76]	Prolgolimab 1 mg/kg 2-weekly	Anti-PD-1	Prolgolimab 3 mg/kg 3-weekly	1st	8.8	40	3.9	16.5	64%	46%	24	8	
2020	Ascierto [2]	Ipilimumab 10 mg/kg 3-weekly	Anti-CTLA-4	Ipilimumab 3 mg/kg 3-weekly	1st/2nd		16		12			7	6	
2018	Long [48]	Nivolumab + ipilimumab	Anti-PD-1/CTLA-4	Nivolumab	1st/2nd	3	9	2	7	46%	20%	9	7	Includes only patients with BM
2016	Gupta [27]	Vandetanib + WBRT	Anti-VEGFR	WBRT + placebo	1st/2nd	3.3	4.6	2.5	2.5			4.6	2.5	Includes only patients with BM

*BM* brain metastasis; *PFS* progression-free survival in months; *OS* overall survival in months; *PD-1* programmed cell death protein 1; *CTLA-4* cytotoxic T-lymphocyte-associated protein 4; *VEGFR*, vascular endothelial growth factor receptor; *WBRT* whole-brain radio therapy

In 2022, Wolchok and collaborators reported long-term results of the CheckMate 067 trial comparing combination therapy of ipilimumab and nivolumab compared to either one alone for disseminated melanoma [82]. These results showed the longest median OS in a phase III melanoma trial reported to date and showed durable, improved clinical outcomes with nivolumab plus ipilimumab. Median OS in the combination arm was 72 months. Brain-specific results of the combination nivolumab plus ipilimumab were reported by Long et Al. [48]. In this smaller phase II trial, 79 patients were enrolled. Intracranial responses were achieved by 46% of the combination cohort compared to 20% with nivolumab alone. Median overall survival was 9 months for the combination group versus 7 for nivolumab monotherapy. These results were better than reported before and set the new standard.

Tawbi et al. reported the long-term outcomes of combination therapy in patients with active melanoma brain metastases treated with combination nivolumab plus ipilimumab (CheckMate 204) [75]. Nivolumab 1 mg/kg plus ipilimumab 3 mg/kg was given intravenously every 3 weeks for four doses, followed by nivolumab 3 mg/kg every 2 weeks for up to 2 years, until disease progression or unacceptable toxicity. One hundred one patients were asymptomatic and 18 were symptomatic. Investigator-assessed intracranial clinical benefit was observed in 57.4% of asymptomatic patients and 16.7% of symptomatic patients. Investigator-assessed objective response was observed in 53.5% patients in asymptomatic and 16.7% in symptomatic patients. For the asymptomatic group, 36-month intracranial PFS was 54.1% and OS 71.9%. For patients in the symptomatic group, 36-month intracranial PFS was 18.9% and OS 36.6%. The incidence of grade 3–4 toxicity was similar to the response rate at 55%.

The current focus of research lies on newer and hopefully more efficient immune checkpoint inhibitors. Tjulandin et al. reported results of the MIRACULUM study, exploring the potential of prolgolimab, an advanced anti-PD-1 (*programmed cell death protein 1)* monoclonal antibody at two different dosing schemes [76]. An objective intracranial response was seen in approximately 50%, and the averaged OS of the patients with brain metastases was 11 months.

Tawbi reported recently early results of relatlimab, a *lymphocyte-activation gene 3*(LAG-3)-blocking antibody, and nivolumab versus nivolumab alone in untreated advanced melanoma (RELATIVITY-047trial) [74]. The median PFS was 10.1 months with relatlimab nivolumab as compared with 4.6 months with nivolumab monotherapy. Thus, the inhibition of two immune checkpoints, LAG-3 and PD-1, provided a greater benefit with regard to progression-free survival than inhibition of PD-1 alone in patients with previously untreated metastatic or unresectable melanoma. So far, brain-specific outcome data are not available.

### Lung cancer

Although pathological classification of lung cancer is quite differentiated and biomarkers that could potentially be used as therapeutic targets are almost limitless, from a practical perspective, subclassification remains straightforward [88]. Some 12–15% are classified as small cell lung cancer (SCLC). For these, no specific molecular targets have been identified, except PD-L1, allowing for immunotherapy, although the predictive value for outcome remains unclear. The remainder of lung cancers, the non-small cell lung cancers (NSCLC), express amplifying EGFR (*epidermal growth factor receptor*) mutations in some 10% and amplifying ALK rearrangements in 4–7%. In addition, during recent years, a number of new and potentially targetable driver mutations have been identified, including BRAF, ROS1 (*c-ros oncogene 1*), EGFR, HER2, RET (*Ret proto-oncogene*), MET (*Met proto-oncogene*), NTRK (*neurotrophic receptor tyrosine kinase 1*), KRAS (*Kirsten RAt sarcoma virus gene*), and others [29, 62].

The pertinent studies on targeted therapy allowing some estimate of treatment effect and the outcome of the subgroup with brain metastases are summarized in Table 3.

**Table 3:** Metastatic lung cancer—pertinent studies allowing some prognostic assessment for patients with brain metastases

Year	Author	Experimental drug/combination	Action	Control drug/combination	1st/ 2nd line	PFS (experimental)	OS (experimental)	PFS (control)	OS (control)	Intracranial response (experimental)	Intracranial response (control)	OS with brain metastasis (experimental)	OS with brain metastasis (control)	Comments
2021	Ma [50]	Atezolizumab	Anti-PD-L1	Docetaxel								16	9	NSCLC, reevaluation POLAR and OAK trials
2021	Horn [31]	Ensartinib	Anti-ALK	Crizotinib	1st/2nd	25.8	> 40	12.7	> 40	64%	21%			NSCLC. ALK +
2020	Shaw [66]	Lorlatinib	Anti-ALK	Crizotinib	1st	> 36	> 36	9.3	> 36	82%	23%			ALK + NSCLC, CROWN trial
2020	Gadgeel [23]	Pembrolizumab + pemetrexed + platinum	Anti-PD-1	Pemetrexed + platinum	1st/2nd	9	22	4.9	10.7			19.2	7.5	NSCLC, KEYNOTE-189 trial, symptomatic BM excluded
2020	Jiang [37]	Anlotinib	Anti-angiogenetic	Placebo	1st/2nd							8.6	4.6	NSCLC, focused on BM, ALTER0303 trial
2019	Huber [33]	Brigatinib 90 mg	Anti-ALK	Brigatinib 180 mg	2nd	9.2	29.5	16.7	34.1	50%	67%	29.5	34.1	NSCLC, ALK + , ALTA trial
2018	Yang [87]	Gefitinib-WBRT	Anti-EGFR	Bevacizumab + gefitinib + WBRT	2nd					70%	80%	10	20	NSCLC, focused on patients with BM
2018	Wu [84]	Osimertinib	Anti-EGFR	Platinum-pemetrexed		11.7		5.6		63%	25%			NSCLC, data from AURA3 trial, only patients with BM
2018	Gadgeel [24]	Alectinib	Anti-ALK	Crizotinib	1st/2nd	9.2		7.4		81%	50%			NSCLC ALK + , ALEX trial, with or wo prev. WBRT
2018	Camidge [10]	Brigatinib	Anti-ALK	Crizotinib	1st					78%	29%			NSCLC, ALK + , ALTA 1L trial,
2018	Camidge [9]	Brigatinib 90 mg	Anti-ALK	Brigatinib 180 mg	2nd	8.8		12.9		53%	67%	> 40	> 20	NSCLC, ALK + , ALTA trial
2017	Yang [86]	Icotinib	Anti-EGFR	WBRT	1st/2nd	6.8	18	3.4	20.5			18	20.5	NSCLC, EGFR mutated with BM, PFS focused on intracranial control
2017	Shaw [67]	Ceritinib	Anti-ALK	Chemotherapy	2nd	5.4	18.1	1.6	20.1	35%	5%			NSCLC ALK + , ASCEND-5 trial
2017	Kim [40]	Brigatinib	Anti-ALK	Different dose	2nd	9.2	> 2 years	12.9	> 2 years	42%	67%			NSCLC ALK + , ca 70% with BM, ALTA trial
2017	Soria [69]	Ceritinib	Anti-ALK, MET, and ROS1	Platinum chemotherapy	1st	16.6	> 35	8.1	30	46%	21%			NSCLC, ALK + , ASCEND 4 trial
2016	Chabot [11]	Veliparib + WBRT	Anti-PARP	Placebo + WBRT	1st/2nd	7.5	7	8.3	6	40%	41%	6.7	6.7	NSCLC with BM
2016	Solomon [68]	Crizotinib	Anti-ALK, MET, and ROS1	Chemotherapy	1st	9		4		77%	28%			NSCLC, ALK + , PROFILE 1014 study, focused on intracranial efficacy, for patients with BM PFS
2015	Besse [7]	Bevacizumab + chemotherapy	Anti-VEGFR		1st	6.7	16			61%		16		NSCLC, with BM, BRAIN trial, WBRT added post trial in most patients
2014	Schuler [64]	Afatinib	Anti-HER family	Chemotherapy	1st	8.2	22.4	5.4	25	73%	24%	22.4	25	NSCLC, sub-analysis from LUX lung 3 and 6, PFS for patients with BM
2014	Wang [78]	gefitinib + WBRT	Anti-VEGFR	VMP chemotherapy + WBRT			13.3		11.7	54%	47%	13.3	11.7	NSCLC with BM
2013	Jiang [38]	Endostatin + WBRT	Anti-VEGFR	WBRT	1st/2nd		10		8			10	8	NSCLC with BM
2012	Gronberg [26]	Enzastaurin + WBRT	Anti-PKC	Placebo + WBRT	1st/2nd		3.8		5.1			3.8	5.1	both SCLC and NSCLC with BM
2011	Pesce [58]	Gefitinib + WBRT	Anti-VEGFR	Temozolomide + WBRT			6.3		4.9			6.3	4.9	NSCLC

*BM* brain metastasis; *PFS* progression-free survival in months; *OS* overall survival in months; *PD-1* programmed cell death protein 1; *PD-L1* programmed cell death ligand 1; *ALK* anaplastic lymphoma kinase; *CTLA-4* cytotoxic T lymphocyte–associated protein 4; *WBRT* whole-brain radio therapy; *VEGF* vascular endothelial growth factor; *VEGFR* vascular endothelial growth factor receptor; *EGFR* epidermal growth factor receptor; *MET* Met tyrosine-protein kinase also known as hepatocyte growth factor receptor (HGFR); *PARP* poly-ADP-ribose-polymerase; *ROS-1* proto-oncogene 1; *PKC* protein kinase C; *SCLC* small cell lung cancer; *NSCLC* non-small cell lung cancer

#### Small cell lung cancer (SCLC)

Specific data regarding intracranial response and survival times are unavailable at the present time for SCLC. Overall prognosis of disseminated SCLC remains poor with overall survival in the range of 6 months. Regarding overall perspective of advanced SCLC, Allen and colleagues reported on a phase II trial of weekly topotecan with and without ziv-aflibercept, a VEGF-trapping agent, in patients with advanced platinum-treated small-cell lung cancer [4]. Overall survival was not significantly improved by addition of ziv-aflibercept (6 versus 4.6 months), but severe toxicities were more common with the addition of ziv-aflibercept.

Based on the negative experience with targeted approaches for SCLC, Morabito and co-workers reported on a trial using either cisplatin plus etoposide at a fixed dose or cisplatin plus etoposide at a variable dose [53]. Seventy percent of patients had no known brain metastases. Response rate was 54.4% and 58.2% in the control and experimental arms, respectively. No significant differences were found in terms of PFS (6 versus 5.6 months) and OS (9.6 versus 9.2 months). The most frequent cause of death was neutropenia with infection. Severe toxicity was more frequent in the experimental arm.

Immune checkpoint inhibitors also raised new hope for SCLC. In 2018, Ready and coworkers reported the results of the CheckMate 032 trial comparing nivolumab monotherapy with a combination of nivolumab plus ipilimumab as second- or third-line treatment in patients with extensive systemic disease [59]. Both arms were comparable: Median PFS with nivolumab monotherapy was 1.4 months and median overall survival 5.6 months.

In the IMpower133 trial, Horn and coworkers reported a substantial benefit by the addition of the PD-L1 blocker atezolizumab to chemotherapy in the first-line treatment of extensive-stage SCLC, which resulted in significantly longer overall survival and progression-free survival than chemotherapy alone [32]. At a median follow-up of 13.9 months, the median OS was 12.3 months in the atezolizumab group and 10.3 months in the placebo group. The median PFS was 5.2 months and 4.3 months, respectively.

The CASPIAN trial confirmed significantly longer overall survival following first-line treatment with the PD-L1 antagonist durvalumab in addition to platinum-based chemotherapy in patients with extensive stage SCLC [56]. Median OS was 13.0 months in the durvalumab plus platinum-etoposide group versus 10.3 months in the platinum-etoposide group.

Owonikoko et al. reported on the effect of the Aurora A kinase inhibitor, alisertib, plus paclitaxel as second-line treatment for SCLC [55]. The median PFS was 3.32 months with alisertib plus paclitaxel versus 2.17 months with placebo plus paclitaxel. Overall survival was 6.1 months versus 5.4 months.

Spigel et al. presented recently the results of Checkmate 331 comparing nivolumab monotherapy with chemotherapy for relapsed SCLC [70]. No significant improvement in OS was seen with nivolumab versus chemotherapy (median OS 7.5 versus 8.4 months). Median progression-free survival was 1.4 versus 3.8 months. Objective response rate was 13.7% versus 16.5% and median duration of response was 8.3 versus 4.5 months. Rates of grade 3 or 4 treatment-related adverse events were 13.8% versus 73.2%.

In summary, perspective for disseminated SCLC remains poor, independent of any CNS involvement.

#### Non-small cell lung cancer (NSCLC)

The abovementioned EGFR and ALK alterations have proved fruitful targets for specific therapy and the overall perspective of these subgroups has markedly improved over the past years. Ten years ago, targeted therapy focused on anti-VEGFR [38, 58, 87]. Overall survival in patients with disseminated disease remained in the range of 6 to 9 months. The introduction of ALK-directed therapy yielded a clear benefit for the ALK + subgroup. In 2016, Solomon and coauthors reported on the intracranial efficacy of first-line crizotinib versus chemotherapy (PROFILE 1014 study) [68]. Patients with stable treated brain metastases were eligible. Twenty-three percent of patients had CNS involvement at baseline. Among these patients, intracranial disease control was significantly higher with crizotinib versus chemotherapy at 12 weeks (85% versus 45%) and at 24 weeks (56% versus 25%). Progression-free survival was significantly longer with crizotinib versus chemotherapy in both subgroups, with brain metastasis at baseline 9.0 versus 4.0 months, and without brain metastasis 11.1 versus 7.2 months.

Further developments brought better efficacy than crizotinib and second-line options after the development of acquired resistance. In a phase II trial in 2017, Kim et al. reported on the efficacy of brigatinib in patients with crizotinib-refractory ALK + NSCLC (ALTA trial) [40]. Patients were stratified by brain metastases and best response to crizotinib. They were randomly assigned to brigatinib 90 mg once daily (arm A) or 180 mg once daily (arm B). Investigator-assessed confirmed objective response rate was the primary endpoint. Seventy percent had baseline brain metastases. Median progression-free survival was 9.2 months and 12.9 months in arms A and B, respectively. Intracranial objective response rate in patients with measurable brain metastases at baseline was 42% in arm A and 67% in arm B. Therefore, brigatinib yielded substantial whole-body and intracranial responses as well as robust progression-free survival; 180 mg showed consistently better efficacy than 90 mg, with acceptable safety.

Further analyses confirmed the superior systemic and intracranial efficacy of brigatinib [9, 10]. Huber et al. focused on the long-term outcome of patients with CNS involvement in the ALTA trial [33]. As mentioned, patients were randomized to brigatinib 90 mg once daily (arm A) or 180 mg once daily with a 7-day lead-in at 90 mg (arm B). At baseline, 71% and 67% had brain lesions among A and B arms, respectively. Objective response rate was 46% versus 56%. Median PFS was 9.2 months versus 16.7 months. Median OS was 29.5 months versus 34.1 months. Intracranial objective response rate in patients with measurable baseline brain lesions was 50% in arm A versus 67% in arm B; median duration of intracranial response was 9.4 versus 16.6 months.

Alectinib, lorlatinib, and ensartinib were further recent developments of anti ALK therapy with superior CNS efficacy. Gadgeel and company reported the results of the ALEX-trial comparing alectinib with crizotinib [24]. In total, 122 patients had CNS metastases and 46 had received prior radiotherapy. CNS objective response rate was 85.7% with alectinib versus 71.4% with crizotinib in patients who received prior radiotherapy and 78.6% versus 40.0%, respectively, in those who had not.

Shaw et al. compared lorlatinib with crizotinib as first-line therapy of advanced ALK + NSCLC [66]. The percentage of patients who were alive without disease progression at 12 months was 78% in the lorlatinib group and 39% in the crizotinib group. An objective response occurred in 76% of the patients in the lorlatinib group and 58% of those in the crizotinib group. Among those with measurable brain metastases, 82% and 23%, respectively, had an intracranial response, and 71% of the patients who received lorlatinib had an intracranial complete response. The most common adverse events with lorlatinib were hyperlipidemia, edema, increased weight, peripheral neuropathy, and cognitive effects. Lorlatinib was associated with more grade 3 or 4 adverse events than crizotinib (72% versus 56%). Discontinuation of treatment because of adverse events occurred in 7% and 9% of the patients, respectively. Horn et al. reported similar systemic and intracranial efficacy of ensartinib compared to crizotinib for advanced ALK + NSCLC [31]. Median PFS was significantly longer with ensartinib than with crizotinib (25.8 versus 12.7 months). The intracranial response rate confirmed by a blinded independent review committee was 63.6% with ensartinib and 21.1% with crizotinib for patients with brain metastases at baseline.

Regarding other targets, Wu et al. reported high CNS efficacy of osimertinib in patients with EGFR T790M mutated advanced NSCLC (AURA3 trial) [84]. Patients with asymptomatic, stable CNS metastases were eligible for enrollment and were randomly assigned 2:1 to osimertinib 80 mg once daily or platinum-pemetrexed. The group evaluable for CNS response included only patients with one or more measurable CNS lesions. Of 419 patients randomly assigned to treatment, 116 had measurable and/or non-measurable CNS lesions, including 46 patients with measurable CNS lesions. CNS objective response rate in patients with one or more measurable CNS lesions was 70% with osimertinib and 31% with platinum-pemetrexed. The objective response rate was 40% and 17%, respectively, in patients with measurable and/or non-measurable CNS lesions. Median CNS duration of response in patients with measurable and/or non-measurable CNS lesions was 8.9 months for osimertinib and 5.7 months for platinum-pemetrexed. Median CNS progression-free survival was 11.7 months and 5.6 months, respectively.

For ALK- and other NSCLC without driver mutation, anti-PD-(L)1 immunotherapy has become the main focus of development. The KEYNOTE-189 trial compared pembrolizumab or placebo plus pemetrexed and platinum for previously untreated metastatic non-squamous NSCLC [23]. Median OS was 22.0 months in the pembrolizumab-combination group versus 10.7 months in the placebo-combination group. Median PFS was 9.0 months and 4.9 months, respectively. OS and PFS benefits with pembrolizumab were observed regardless of PD-L1 expression or presence of liver/brain metastases. Overall survival in the subgroup with brain metastases was 19.2 months with pembrolizumab versus 7.5 months.

In summary, for the patients with intracranial dissemination of NSCLC, intracranial response rates of up to some 80% and overall survival of up to 2 years are reported for ALK + entities with intracranial dissemination. Anti-PD-1 immunotherapy appears to be also promising for ALK + and ALK- varieties with intracranial dissemination, but data are currently not yet sufficient, and the question of combination immunotherapeutic and addition of chemotherapy remains unsolved for NSLC without driver mutation.

## Discussion

Reviewing the currently available data unveiled a clear deficit of information regarding cancer patients with very advanced stage of disease, i.e., patients suffering of intracranial dissemination. In the majority of pharmacological studies, patients suffering of intracranial dissemination were excluded a priori. In the others, inclusion is limited to variably defined stable disease. Interpretation is further hindered by the lack of information regarding adjuvant radiotherapy.

The reviewed data suggest that introduction of targeted therapies brought a clear improvement of survival for the subgroups of cancers expressing an addressable target. For example, according to our analysis and the meta-analysis of Cameron et al., for the 4–7% of ALK + NSCLC, introduction of ALK inhibitors resulted in a large increase in PFS and increase of ORR including patients with measurable baseline brain metastases when compared to chemotherapy [8]. ALK inhibitors improved OS to a lesser degree than PFS.

The introduction of next-generation ALK inhibitors alectinib, brigatinib, and lorlatinib again resulted in a clear improvement of PFS and ORR compared to the first generation ALK inhibitor crizotinib, particularly in participants with baseline brain metastases. Next-generation inhibitors likely improve also OS to some degree.

In the case of NSCLC exhibiting EGFR mutations, our analysis confirms the meta-analysis of Erickson, Brastianos, and Das, that only introduction of osimertinib brought measurable progress regarding PFS and objective response rate, while effects of other substances are minimal for the case of advanced disease with brain metastases [21].

Generally, the new targeted therapies for advanced cancer often appear also to benefit patients with brain metastases. However, more or less solid evidence is only available for breast and lung cancer and melanoma. Targeted therapies have become accepted for other types of cancer; i.e., tyrosine kinase inhibitors and immune checkpoint inhibitors are a firm part of adjuvant first- or second-line therapy of renal cancer, but so far, there are no data available that allow an estimate of intracranial efficacy [54].

Intracranial escape of metastases due to the blood–brain barrier is a well-known problem in cancer therapy [41]. Intracranial metastases may respond to targeted therapy worse than extracranial dissemination. Therefore, in the absence of data showing intracranial efficacy, we cannot just substitute results of extracranial efficacy.

Further problems are potential differences between primary tumor profiles and metastasis, e.g., hormone receptors in breast cancer, where conversion of estrogen and progesterone receptors is not uncommon. Stereotactic biopsy could potentially clarify variations between primary and metastasis. However, appreciating the clinical importance is difficult, since we do not have any substantial data on the efficacy of anti-hormone therapy for brain metastases from breast cancer. Taken these aspects together and in view of the risk of stereotactic biopsy, including tumor seeding, we believe that at the moment, there is no indication for brain biopsy with the aim to clarify receptor or mutational status of intracranial metastases.

Radiotherapy, surgery, and radiosurgery have also achieved significant progress during the last decades [25, 61]. One perspective could be that control of intracranial manifestation can today be achieved by possibly repeated radiosurgery [52]. This would lead to the view that intracranial efficacy of the pharmaceutical adjuvant therapy is of lesser importance. As of today, this approach is certainly the pragmatic one for cancer types with missing data regarding intracranial efficacy of adjuvant pharmacological therapy. The combination of radiosurgery with targeted therapies particularly BRAF inhibitors may increase the risk of radionecrosis [42] and the optimal sequencing of such combined-modality therapy is the subject of numerous open clinical trials.

Although directed therapy for the cancer subgroups with an appropriate target has an effect, the latter is still limited in extent and duration. Anti-ALK-directed therapy of ALK + lung cancers does not lead to the disappearance of these cells. Moreover, the duration of tumor control appears to be generally limited, and after a certain time tumors find escape mechanisms and then progress again. In that respect, targeted therapy does not differ from traditional chemotherapy and both modalities act on vital cellular pathways, which are also important for normal cells. Completely blocking these pathways would also kill normal cells.

As mentioned earlier, firm data on the brain-specific effect of targeted therapies are only available for the most common cancers. For the others, we have no good evidence and have to estimate the potential effect of targeted therapies based on the effect on the primary tumor or on the basis of non-randomized studies, as, for example, in the case of renal cell carcinoma [35]. Data pertaining to the prognosis of the different subgroups are also lacking. Data regarding the efficacy on intracranial dissemination are particularly scarce for the more benign tumor variants. For example, the multikinase blocker palbociclib is used in combination with endocrine therapy for hormone receptor–positive breast cancer [77], but we do not have solid information on the efficacy in the case of intracranial dissemination [45]. As the case report of Abused and colleagues shows, it may be very effective [1].

Current targeted approaches address only a few of many possible pathways. Reviewing the last decade, identification and exploration of new targets appears to be accelerating. At the time of writing, new preliminary data from the TUXEDO-1 trial suggest high intracranial response rates of trastuzumab-deruxtecan in HER2-positive breast cancer patients with active brain metastases, with a progression free survival of 14 months [6]. With the advent of new potential targets and drug combinations, clinical validation will be a major challenge.

Although current targeted therapies have advanced, the gain for patients with cancer disseminated to the brain remains limited. It appears therefore particularly important to focus in the future also on the quality of life during the last months of life.

## Conclusion

In conclusion, the introduction of targeted therapy roughly doubled survival times of many advanced cancers including those metastasized to the brain. Currently only few cell signal pathways have been targeted, i.e. pathways that are also important for the healthy cells. Therefore today’s targeted therapy does not differ essentially from chemotherapy, in that it hurts both cancer and healthy cells, only with some stronger effect on the cancer cells. However, there are many more potential molecular targets, and the hope remains that targets can be found that are much more specific for cancer cells than the ones so far explored. Further clinical trials will also have to address quality of live in addition to pure length of survival.

## Data Availability

Not applicable.
